# Effects of a reminiscence therapy-involved program on anxiety, depression, and the quality of life in cancer patients: a meta-analysis of randomized controlled trials

**DOI:** 10.3389/fpsyg.2024.1408941

**Published:** 2024-09-17

**Authors:** Xingliang Sun, Wenlian Su, Mengyue Yin, Ling Xia

**Affiliations:** Department of Rehabilitation Medicine, Zibo Central Hospital, Zibo, China

**Keywords:** reminiscence therapy-involved program, cancer patients, anxiety and depression, quality of life, meta-analysis

## Abstract

**Objective:**

Reminiscence therapy is increasingly being utilized for cancer patients to address psychological pressure and enhance their quality of life. This meta-analysis aimed to comprehensively evaluate the effect of a reminiscence therapy-involved program (RTIP) on anxiety, depression, and quality of life in cancer patients.

**Methods:**

A systematic literature search was conducted in the Web of Science, PubMed, Embase, and Cochrane Library databases until December 2023 to screen randomized control trials (RCTs) comparing the effect of RTIP and control care.

**Results:**

A total of 16 RCTs published from 2013 to 2023 were included, with 1,963 cancer patients undergoing RTIP with or without control care (RTIP group, *N* = 984) or control care (control group, *N* = 979). The results showed the the anxiety score [standardized mean differences (SMD) = −0.539; 95% confidence interval (CI) = −0.700, −0.378; *P* < 0.001], anxiety rate [relative risk (RR) = 0.736; 95% CI: 0.627, 0.865; *P* < 0.001], depression score (SMD = −0.664; 95% CI: −0.967, −0.361; *P* < 0.001), and depression rate (RR = 0.632; 95% CI = 0.532, 0.750; *P* < 0.001) were significantly reduced in the RTIP group compared to the control group. Furthermore, overall quality of life was increased in the RTIP group than in the control group (SMD = 0.501; 95% CI: 0.314, 0.689; *P* < 0.001). In digestive system cancer patients, anxiety/depression scores and rates were reduced, and the overall quality of life was elevated in the RTIP group in comparison with the control group (all *P* < 0.050). The quality of evidence was generally high, with a low risk of bias in most studies and no publication bias in any outcomes (all *P* > 0.050).

**Conclusion:**

RTIP attenuates anxiety and depression and improves the quality of life in cancer patients, benefitting their overall health condition.

**Systematic Review Registration:**

This meta-analysis was registered at PROSPERO with registration number CRD42024563266.

## 1 Introduction

Cancer is one of the leading obstacles to increasing life expectancy, with ~19.9 million new cases and 9.7 million deaths worldwide in 2022 (Sung et al., 2021; Bray et al., 2024). Conceivably, the psychological burden of cancer patients is heavy, caused not only by confronting major life stressors or threats but also by many other factors, including the physical dimension, treatment dimension, as well as economic and interpersonal communication aspects (Emery et al., 2022). For the physical dimension, it is estimated that 32%−90% of patients experience cancer-related fatigue, and 35%−96% of patients suffer from cancer-related neuropathy or bone pain, which enhances psychological pressure (Henson et al., 2020; Renna et al., 2022; Wu et al., 2022). Concerning the treatment dimension, postoperative complications and medication/radiotherapy-induced side effects lead to increased perceived pressure and a sense of loss in cancer patients (Wagland et al., 2015; Henson et al., 2020). Moreover, financial burdens and social isolation aggravate the psychological pressure of cancer patients (Abrams et al., 2021; Liang et al., 2022). More importantly, the heavy psychological burden, commonly manifested by anxiety and depression, is linked with treatment discontinuation, disease recurrence, and death of cancer patients (Morrison et al., 2017; Wang et al., 2020). In addition, an unpleasant quality of life frequently occurs in cancer patients, which is adversely affected by the aforementioned influencing factors and psychological pressure as well (van Montfort et al., 2020; Carbajal-Lopez et al., 2022; Jiang et al., 2023; Licu et al., 2023). Studies have reported that poor quality of life and anxiety and depression are prevalent in patients with cancers (Zheng et al., 2020; Li Y. et al., 2022; Fu et al., 2023). Consequently, developing oncology care plans and providing proper intervention to help cancer patients cope with psychological pressure as well as improve their quality of life is quite necessary (Mullen et al., 2023).

Reminiscence therapy, developed by Robert Butler in 1963 and initially applied in the elderly population with cognitive impairment, is a life-reviewing caring approach that encourages subjects to recall autobiographical events (Cuevas et al., 2020; Yan et al., 2023). This intervention has been widely used in patients with cognitive impairment, such as Alzheimer's disease, post-stroke cognitive impairment, and dementia (Smallfield et al., 2024). During the intervention procedure, it was surprisingly noticed that reminiscence therapy can relieve psychological pressure and enhance the self-confidence of participants (Tam et al., 2021). Gradually, reminiscence therapy has been introduced in cancer patients to alleviate anxiety/depression and improve quality of life, which has served as optional management to improve the lives of patients with cancers (Xiao et al., 2013; Vuksanovic et al., 2017; Kleijn et al., 2018; Dong et al., 2019; Liu and Li, 2021; Zhang et al., 2021; Zhao, 2021; Zhou and Sun, 2021; Chen et al., 2022; Guo et al., 2022; Huang et al., 2022; Li T. et al., 2022; Liu et al., 2022; Zheng et al., 2022; Babaei et al., 2023; Wu and Zhang, 2023). For instance, one study discloses reduced anxiety score, anxiety rate, and depression score, as well as elevated quality of life in colorectal cancer patients who receive reminiscence therapy compared to those treated with control care, while the depression rate is similar between them (Zhou and Sun, 2021). Another study shows that reminiscence therapy decreases anxiety score and anxiety rate, but it does not affect depression score or depression rate in surgical gastric cancer patients (Zhang et al., 2021). Differently, one previous study indicates that no difference is observed in anxiety score, depression score, or quality of life between cancer patients undergoing reminiscence therapy and usual care (Kleijn et al., 2018).

To date, only one previous meta-analysis has indicated that reminiscence therapy ameliorates anxiety and depression and improves the quality of life in cancer patients (Sun et al., 2023). However, the aforementioned meta-analysis includes articles from 2010 to 2021 (Sun et al., 2023), and a number of relevant studies (*n* = 8) published after 2021 have not yet been included in a pooled analysis (Chen et al., 2022; Guo et al., 2022; Huang et al., 2022; Li Y. et al., 2022; Liu et al., 2022; Zheng et al., 2022; Babaei et al., 2023; Wu and Zhang, 2023).

In this study, this meta-analysis summarized the existing randomized control trials (RCTs), intending to comprehensively evaluate the effect of a reminiscence therapy-involved program (RTIP) on anxiety, depression, and the quality of life in cancer patients.

## 2 Methods

### 2.1 Database searching

This meta-analysis has been registered at PROSPERO with registration number CRD42024563266. Two investigators systematically and independently searched English databases, including Web of Science, PubMed, Embase, and Cochrane Library. The study's search time span was from the establishment to December 2023. The search terms contained “reminiscence therapy,” “life review therapy,” “cancer,” “tumor,” and “malignant neoplasm.”

### 2.2 Study selection

The inclusion criteria for this meta-analysis were as follows: (1) studies reported comparisons between RTIP and control intervention in cancer patients; (2) studies reported patients age were more than 18 years old; (3) studies reported data about physiological pressure (anxiety or depression) or quality of life; (4) studies were RCTs; and (5) studies were published in English. The exclusion criteria were as follows: (1) duplicative studies; (2) studies did not contain relevant data that could be extracted or relevant data could not be used; (3) reviews, meta-analyses, or case reports; and (4) studies reported data in cancer patients' caregivers. Two investigators completed this part of the study, and in case of disagreement, they discussed the decision with a third researcher.

### 2.3 Definition and grouping

The control intervention contained blank, health education, exercise guidance, routine care, usual care, control care, and so on. The “control” group was defined as cancer patients who received control intervention. The RTIP was defined as life review therapy or reminiscence therapy, through shared memories or past experiences, aimed at improving the patient's physiological pressure and overall quality of life. The “RTIP” group was defined as cancer patients who underwent the RTIP with or without control intervention.

### 2.4 Quality assessment

After determining the included studies, two investigators read the studies independently. The quality of the studies was assessed via the Cochrane Collaboration Risk of Bias Assessment Instrument (Zeng et al., 2015). If the study met the criteria, it indicated that the occurrence of various biases was minimal (low). If the quality criteria were partially satisfied, it suggested that the possibility of bias occurrence was moderate or unclear (unclear). However, if the study did not meet the criteria, it implied a high risk of bias (high). For the overall assessment, if all items were low, the overall result was low; if one or more items were assessed as high, the overall result was high, and the remaining cases were considered unclear.

### 2.5 Data extraction

The first author's name, publish year, sample size, cancer type, age, sex, intervention-related information, and ending index were extracted by two investigators. The extracted ending indexes included anxiety score [measured by Patients Dignity Inventory (PDI), hospital anxiety or depression scale (HADS) for anxiety, or Self-Rating Anxiety Scale (SAS)], anxiety rate, depression score [measured by PDI, HDAS for depression, Self-Rating Depression Scale (SDS), or Beck's Depression Inventory], depression rate, and overall quality of life [measured by overall quality of life, European Organization for Research and Treatment of Cancer Quality-of-Life Questionnaire PAL 15 (EORTC QLQ-C15-PAL), or EORTC QLQ-Core 30]. For the studies with only relevant figure results, we first tried to contact the corresponding authors to seek the original data. If this was not successful, a gadget called “GetData” was used to extract values from the figures.

### 2.6 Data analysis

RStudio software based on R version 4.3.1 was used to analyze data. The standardized mean differences (SMD) with a 95% confidence interval (CI) were used to analyze the continuous variable and eliminate the differences between scales (Schwarzer et al., 2015). The relative risk (RR) with a 95% CI was adopted for dichotomous outcomes. Heterogeneity across studies was determined via the *I*^2^ test and *Q* test. The random effects model was used when the heterogeneity existed. Publication bias was assessed using Egger's test. Besides, studies reported that patients with digestive system cancer accounted for a large proportion of the population, so those studies with the above particular populations were extracted separately for meta-analysis. A *P* value < 0.05 indicated significance.

## 3 Results

### 3.1 The procedure of study selection

**Figure 1:**
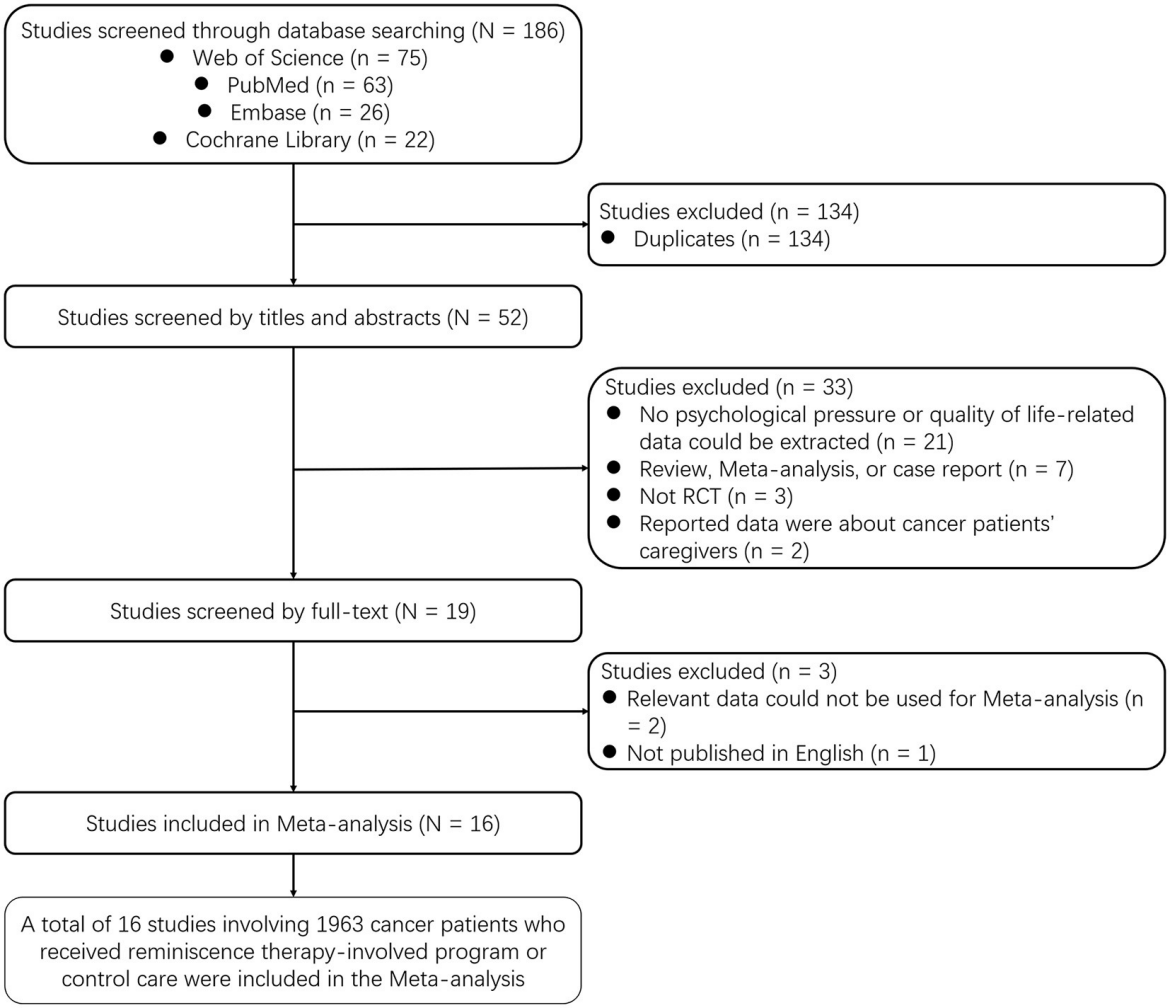
Study flow.

A total of 186 studies were screened in the Web of Science (*n* = 75), PubMed (*n* = 63), Embase (*n* = 26), and Cochrane Library (*n* = 22) databases. Among them, 134 duplicated studies were excluded. Then, the remaining 52 studies were screened by titles and abstracts. During this process, 33 studies were excluded, including 21 studies with no psychological pressure or quality of life-related data, seven review/meta-analysis/case reports, three non-RCTs, and two studies focusing on cancer patients' caregivers. Subsequently, 19 studies were screened in full-text, and three of them were excluded for relevant data inapplicable for meta-analysis (*n* = 2) and not published in English (*n* = 1). Finally, 16 studies involving 1,963 cancer patients who underwent RTIP or control care were included in this meta-analysis (Figure 1).

### 3.2 Information on the enrolled studies and treatment

The included 16 RCTs were published from 2013 to 2023, containing 1,963 cancer patients who underwent RTIP with or without control care (*N* = 984) or control care (*N* = 979) (Xiao et al., 2013; Vuksanovic et al., 2017; Kleijn et al., 2018; Dong et al., 2019; Liu and Li, 2021; Zhang et al., 2021; Zhao, 2021; Zhou and Sun, 2021; Chen et al., 2022; Guo et al., 2022; Huang et al., 2022; Li Y. et al., 2022; Liu et al., 2022; Zheng et al., 2022; Babaei et al., 2023; Wu and Zhang, 2023). The involved cancer types included colorectal cancer, lung cancer, gastric cancer, glioma, papillary thyroid carcinoma, prostate cancer, hepatocellular carcinoma, and cervical cancer. The details of the included studies are exhibited in Table 1. In addition, the intervention-related information, including types of intervention, sessions of intervention, and practice approach, is listed in Table 2.

**Table 1:** Features of the included studies.

**References**	**Sample size**		**Cancer type**	**Age (years)**		**Female**		**Measurement tool**	**Ending index**
	**RTIP group**	**Control group**		**RTIP group**	**Control group**	**RTIP group**	**Control group**		
Xiao et al. (2013)	40	40	Polytypic	58.5 ± 11.8	59.8 ± 11.3	50.0%	45.0%	Overall quality of life	⑤
Vuksanovic et al. (2017)	18	18	Polytypic	62.3 ± 16.2	54.9 ± 13.8	50.0%	61.1%	PDI	①, ③
Kleijn et al. (2018)	55	52	Polytypic	64.2 ± 8.5	61.2 ± 9.9	47.3%	46.2%	HADS-A, HADS-D, EORTC QLQ-C15-PAL	①, ③, ⑤
Dong et al. (2019)	45	45	Colorectal cancer	<50: 8.9% 50–60: 33.3% 60–70: 42.2% >70: 15.6%	<50: 8.9% 50–60: 37.8% 60–70: 46.7% >70: 6.7%	46.7%	51.1%	SAS, SDS	①, ③
Zhou and Sun (2021)	105	105	Colorectal cancer	64.0 ± 10.8	64.5 ± 9.6	42.9%	45.7%	HADS-A, HADS-D, EORTC QLQ-Core 30	①, ②, ③, ④, ⑤
Liu and Li (2021)	96	96	Non-small cell lung cancer	61.0 ± 10.1	60.0 ± 9.6	27.1%	25.0%	HADS-A, HADS-D, EORTC QLQ-Core 30	①, ②, ③, ④, ⑤
Zhang et al. (2021)	80	80	Gastric cancer	59.1 ± 10.9	60.4 ± 9.9	55.0%	52.5%	HADS-A, HADS-D, EORTC QLQ-Core 30	①, ②, ③, ④, ⑤
Zhao (2021)	75	75	Glioma	48.7 ± 10.6	50.8 ± 11.3	38.7%	44.0%	HADS-A, HADS-D	①, ②, ③, ④
Chen et al. (2022)	44	42	Papillary thyroid carcinoma	64.3 ± 3.3	65.5 ± 3.4	68.2%	64.3%	HADS-A, HADS-D, EORTC QLQ-Core 30	①, ②, ③, ④, ⑤
Guo et al. (2022)	69	69	Lung cancer	68.3 ± 5.2	68.2 ± 4.3	18.8%	20.3%	HADS-A, HADS-D	①, ②, ③, ④
Huang et al. (2022)	55	53	Prostate cancer	62.4 ± 9.0	63.1 ± 9.4	0.0%	0.0%	HADS-A, HADS-D, EORTC QLQ-Core 30	①, ②, ③, ④, ⑤
Li T. et al. (2022)	52	54	Hepatocellular carcinoma	68.0 ± 5.4	68.7 ± 5.5	14.8%	11.5%	HADS-A, HADS-D, EORTC QLQ-Core 30	①, ②, ③, ④, ⑤
Liu et al. (2022)	76	76	Cervical cancer	49.8 ± 9.9	52.2 ± 11.3	100.0%	100.0%	HADS-A, HADS-D, EORTC QLQ-Core 30	①, ②, ③, ④, ⑤
Zheng et al. (2022)	50	50	Digestive system cancer	57.5 ± 9.3	58.5 ± 10.0	26.0%	41.0%	HADS-A, HADS-D	①, ③
Babaei et al. (2023)	76	76	Gastric cancer	62.0	62.0	34.2%	34.2%	SAS, Beck's Depression Inventory	①, ③
Wu and Zhang (2023)	48	48	Gastric cancer	60.9 ± 10.7	57.4 ± 11.9	39.6%	27.1%	HADS-A, HADS-D, EORTC QLQ-Core 30	①, ②, ③, ④, ⑤

RTIP, reminiscence therapy-involved program; PDI, Patients Dignity Inventory; HADS-A, hospital anxiety or depression scale for anxiety; HADS-D, hospital anxiety or depression scale for anxiety; EORTC QLQ-C15-PAL, European Organization for Research and Treatment of Cancer Quality-of-Life Questionnaire PAL 15; EORTC QLQ-Core 30, European Organization for Research and Treatment of Cancer Quality-of-Life Questionnaire Core 30.

The data of “age” were shown by mean ± standard deviation, percentage, or mean value.

①: anxiety score; ②: anxiety rate; ③: depression score; ④: depression rate; and ⑤: overall quality of life.

**Table 2:** Intervention-related information.

**References**	**Types of intervention**		**Sessions of intervention**		**Practice approach**		**Details of RTIP**	
	**RTIP group**	**Control group**	**RTIP group**	**Control group**	**RTIP group**	**Control group**	**Contents**	**Facilitators**
Xiao et al. (2013)	LR program plus routine care	Routine care	3	3	Home-based	Home-based	1st session: focused on reviewing the present life; 2nd session: focused on adulthood; 3rd session: reviewed the patient's childhood and adolescence	Nurse
Vuksanovic et al. (2017)	LR protocol	Blank	(–)	(–)	Hospital-based	(–)	The intervention was completed in a conversation that was determined by the participant according to the dignity therapy question framework	Psychologist
Kleijn et al. (2018)	LR plus memory specificity training	Blank	4	(–)	Home-based	(–)	Four sessions on a particular lifetime period: childhood, adolescence, adulthood, and whole life span.	Psychologist
Dong et al. (2019)	RT	Usual care	6	6	Telephone-based	(–)	1st session: The psychologist guided the patient to reminisce about people who had a positive influence on their lives; 2nd session: The patient reminisced about happy times in their past; 3rd session: The patient talked about his or her past achievements and the significance of these achievements; 4th session: The patient recalled the important turning points in his or her life and the influence of each; 5th session: The patient talked about his or her struggles with cancer and its positive significance. 6th session: The patient talked about his or her hopes for the future	Psychologist
Zhou and Sun (2021)	RT plus health education and exercise guidance	Health education and exercise guidance	24	24	Hospital-based and home-based	Hospital-based and home-based	Topic 1: Introducing yourself and a brief family history; Topic 2: Sharing your childhood memories; Topic 3: Sharing school life and memories; Topic 4: Sharing the memories of wooing and marriage; Topic 5: Sharing career experiences and achievements; Topic 6: Sharing personal hobbies and showing your achievements about hobbies; Topic 7: Elaborating a decisive event in one's life; Topic 8: Sharing your individual photos and telling their story; Topic 9: Sharing friends' stories; Topic 10: Sharing memory of hometown; Topic 11: Talking about Chinese opera, old movies or songs; Topic 12: Reviewing overall 24 sessions and farewell	Nurse
Liu and Li (2021)	RT plus health education and aerobic exercise guidance	Health education and aerobic exercise guidance	24	24	Hospital-based	Hospital-based	12 scheduled topics: (1) introducing yourself and brief family history; (2) sharing childhood memories and favorite games; (3) sharing school life and memories related to adolescence and youth; (4) sharing the memories of wooing and marriage; (5) sharing career experiences and achievements; (6) highlighting roles of the individuals at home and corporation; (7) elaborating a decisive event in one's life (a decisive event was an experience that one had in his or her life leading to a major change in life); (8) sharing your individual photos and telling their story; (9) sharing friend stories; (10) sharing memory of hometown; (11) talking about Chinese opera, old movies, or songs; (12) reviewing overall 24 sessions and farewell	Nurse
Zhang et al. (2021)	RT plus usual care	Usual care	24	24	Hospital-based	Hospital-based	Introducing yourself and sharing a brief family history; sharing childhood stories; sharing school life stories; sharing the memory of hometown; introducing the custom of Spring Festival in your hometown; sharing love experience and married life; sharing career and work experience; sharing an adventure experience; elaborating an epoch-making event in one's life; sharing old photos or videos and related stories; showing our talents; reviewing 24 sessions and saying goodbye to each other	Nurse
Zhao (2021)	RT plus control care	Control care	24	24	Hospital-based	Hospital-based	(1) Introduce yourself and brief your family history; (2) talk about childhood memories and favorite games; (3) share school life and memories related to adolescence and youth; (4) talk about the memories of wooing and marriage; (5) sharing career experiences and achievements; (6) highlighting roles of the individuals at home and corporation; (7) elaborating on a decisive event in one life (a decisive event was an experience that one had in his or her life leading to a major change in life); (8) sharing your individual photos and telling their story; (9) sharing friend stories; (10) sharing a memory of hometown; (11) talking about Chinese opera, old movies, or songs; (12). reviewing overall 24 sessions and farewell	Nurse
Chen et al. (2022)	RT plus usual care program	Usual care program	12	12	Hospital-based	Hospital-based	(a) a brief self-introduction; (b) sharing an interesting childhood story; (c) sharing a memorable school story; (d) sharing the scenery of the hometown; (e) sharing the favorite food of the hometown; (f) sharing a memorable travel experience; (g) sharing professional experiences; (h) sharing a personal hobby; (i) sharing a favorite sport; (j) sharing a favorite celebrity or star; (k) sharing a favorite book or music; and (l) review and summarization	(–)
Guo et al. (2022)	RT-involved care program plus usual care program	Usual care program	24	24	Hospital-based	Hospital-based	(1) introduction of oneself and a brief family history, (2) sharing funny things from childhood, (3) sharing stories from school life, (4) sharing memories of wooing and marriage, (5) sharing special customs of their hometown, (6) sharing career experiences, (7) sharing an unforgettable travel experience, (8) sharing your favorite movies or songs, (9) sharing personal hobbies and showing your hobby-related achievements, (10) sharing your favorite historical personage and their legendary story, (11) participating in a talent show, and (12) review and summary	Nurse
Huang et al. (2022)	RT plus usual care program	Usual care program	24	24	Hospital-based	Hospital-based	(1) self-introductions of personalities and their family; (2) funny things in childhood; (3) school life stories; (4) memory of hometown; (5) festival customs in your hometown; (6) romantic experiences and marriage life; (7) working experiences; (8) unforgettable travel experience; (9) an epoch-making event in one's life; (10) favorite movie or songs; (11) talent show; (12) summary and farewell	Nurse
Li T. et al. (2022)	RT plus control care program	Control care program	12	12	Hospital-based	Hospital-based	(1) introducing yourself and your family; (2) sharing the memories of childhood; (3) recalling stories from your school days; (4) sharing the memories of marriage; (5) sharing unique customs of your hometown; (6) sharing your work experiences; (7) sharing an interesting trip; (8) sharing an event that affected the course of your life; (9) sharing personal hobbies; (10) sharing your favorite heroes and their legends; (11) individual talent show; and (12) review and farewell	Researchers
Liu et al. (2022)	RT plus routine care	Routine care	24	24	Hospital-based	Hospital-based	(1) introducing yourself and your family; (2) sharing a childhood story; (3) sharing a school life story; (4) introducing your hometown; (5) introducing a story happened in the Spring Festival; (6) sharing your romantic experiences; (7) sharing your career story; (8) sharing a memorable experience; (9) sharing your favorite movie; (10) sharing old photos and the related stories; (11) showing your hobbies or interests; (12) review, summary, and farewell	Nurse
Zheng et al. (2022)	LR program plus routine care	Routine care	4	4	WeChat-based	(–)	Interview on WeChat: present life (cancer experience); adulthood; childhood and adolescence; and summary of life. Asynchronous communication: Memory prompts, Review extraction, Mind space, and E-legacy product	Nurse
Babaei et al. (2023)	RT plus usual care	Usual care	6	6	Hospital-based	Hospital-based	week 1: The psychiatric nurse helped the patients recall their memories of people with positive effects on their lives; week 2: The patients told memories of happy moments in their past; week 3: The patients talked about their achievements and their significance; week 4: The patients recalled significant turning points in their lives and how they were affected by them; week 5: the patients talked about their struggles with cancer and the importance of these struggles and their positive effects; week 6: The patients talked about their hopes for the future	Psychiatric nurse
Wu and Zhang (2023)	RT plus usual care	Usual care	12	12	Hospital-based	Hospital-based	Self-introduction and a brief outline of your family; sharing childhood memories; sharing campus life; sharing memories of marriage (memories of love for patients not married); sharing unique traditions of your homeland; sharing the stories in your career (the stories of teamwork for patients who had not been employed); sharing a memorable travel experience; sharing your best-loved movie or songs; sharing your personal leisure pursuit; sharing your best-loved historical figure and their well-known legend; talent show; review and summarization	Nurse

RTIP, reminiscence therapy-involved program; LR, life review; RT, reminiscence therapy; (–), no information provided.

### 3.3 Risk of bias and publication bias

**Figure 2:**
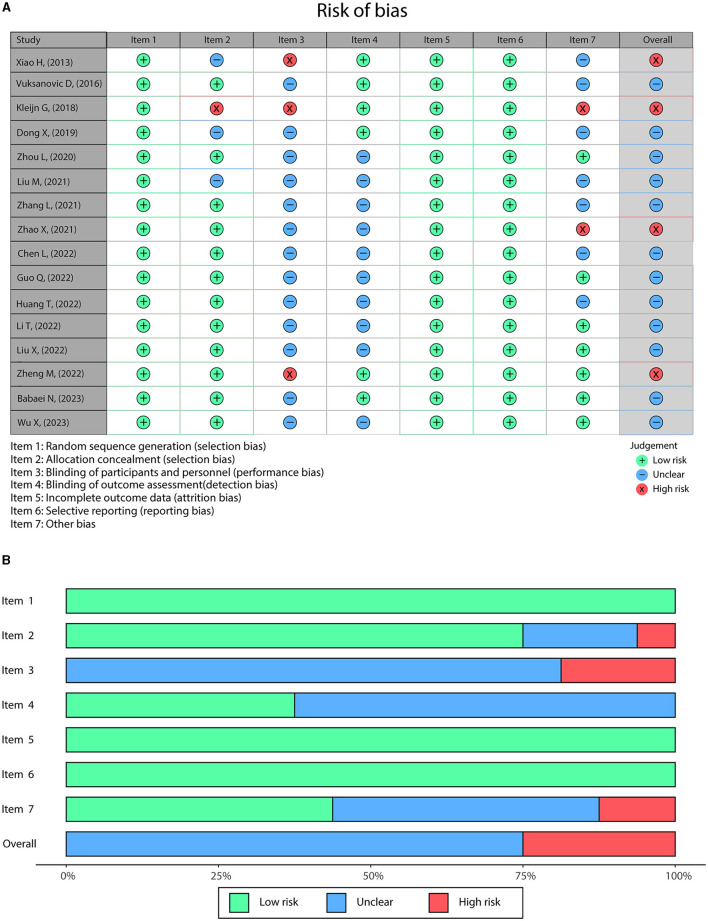
The risk of bias was evaluated using standard Cochrane criteria. Detailed **(A)** and overall **(B)** risk of bias in the included studies.

Overall, most articles were conducted with rigorous randomization (16/16), allocation concealment (12/16), complete outcome data (16/16), and low risk of selective reporting (16/16), while the performance bias (13/16) and detection bias (10/16) were unclear in most studies. Besides, a high risk of bias arose in four articles, containing one study at high risk of performance bias (Xiao et al., 2013), one study at high risk of selection bias, performance bias, and other bias (Kleijn et al., 2018), one study at high risk of other bias (Zhao, 2021), and one study at high risk of performance bias (Zheng et al., 2022) (Figures 2A, B). The Rob 2.0 tool was also applied to assess the quality of the studies (Supplementary Table 1). The results showed that all studies were considered low risk regarding bias arising from the randomization process, bias due to missing outcome data, and bias in selecting the reported result. Moreover, 13 studies were assessed as low risk regarding bias due to deviations from intended interventions, while the other three studies had some concerns; six studies were considered low risk regarding bias in measuring the outcome, while the other 10 had some concerns.

Egger's test disclosed that no publication bias existed in each outcome, including anxiety score, anxiety rate, depression score, depression rate, and overall quality of life (all *P* > 0.050; Table 3).

**Table 3:** Publication bias.

**Items**	**Egger's test**
Anxiety score	0.972
Anxiety rate	0.605
Depression score	0.183
Depression rate	0.141
Overall quality of life	0.094

### 3.4 Effect of RTIP on anxiety

**Figure 3:**
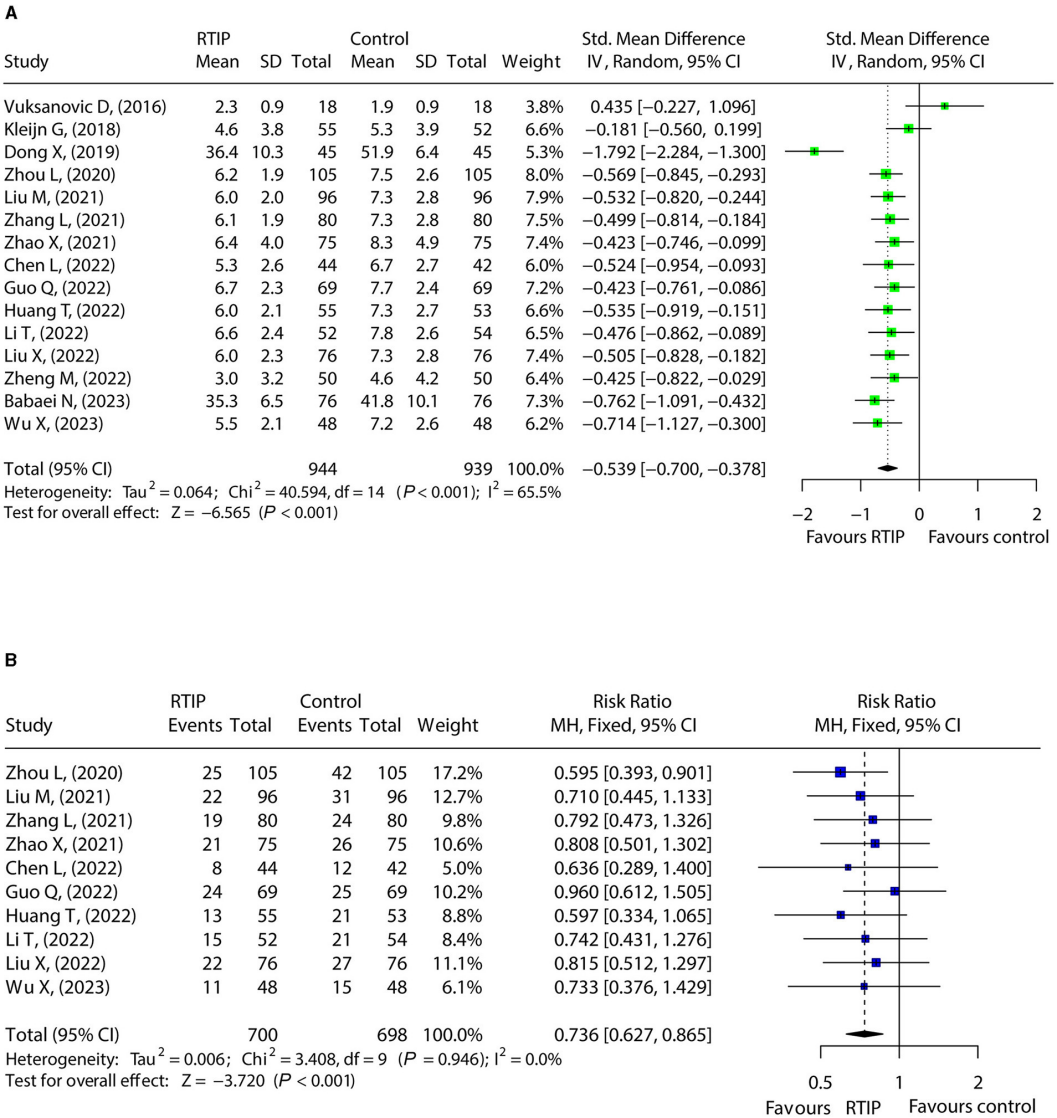
RTIP with or without control care reduced anxiety in cancer patients. Forest plot for the effect of RTIP on anxiety score **(A)** and anxiety rate **(B)** in cancer patients.

Fifteen studies compared anxiety scores between the RTIP and control groups with heterogeneity (*I*^2^ = 65.5%, *P* < 0.001). The random effects model showed that anxiety score was reduced in the RTIP group compared to the control group [SMD (95% CI): −0.539 (−0.700, −0.378), *P* < 0.001; Figure 3A]. Besides, 10 studies compared anxiety rates between the RTIP and control groups, and there was no heterogeneity (*I*^2^ = 0.0%, *P* = 0.946). Pooled analysis revealed that the anxiety rate declined in the RTIP group compared to the control group [RR (95% CI): 0.736 (0.627, 0.865), *P* < 0.001; Figure 3B].

### 3.5 Effect of RTIP on depression

**Figure 4:**
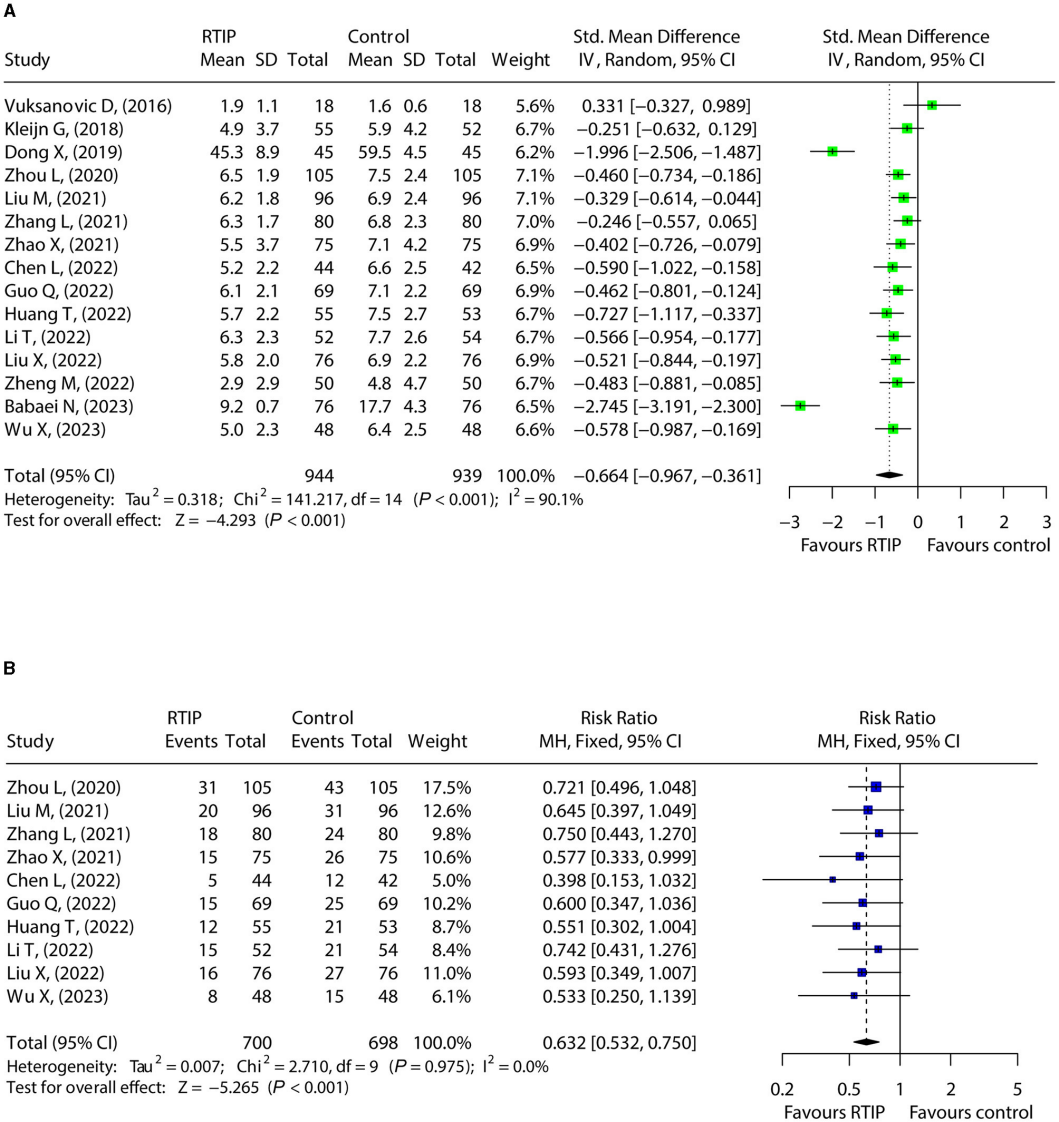
RTIP with or without control care reduced depression in cancer patients. Forest plot for the effect of RTIP on depression score **(A)** and depression rate **(B)** in cancer patients.

A total of 15 studies compared depression scores between the RTIP group and the control group. Data were heterogeneous (*I*^2^ = 90.1%, *P* < 0.001). The depression score declined in the RTIP group compared with the control group [SMD (95% CI): −0.664 (−0.967, −0.361), *P* < 0.001; Figure 4A]. A total of studies reported depression rates in the RTIP group and control group without heterogeneity (*I*^2^ = 0.0%, *P* = 0.975). After pooled analysis, it was found that the depression rate was lower in the RTIP group compared to the control group [RR (95% CI): 0.632 (0.532, 0.750), *P* < 0.001; Figure 4B].

### 3.6 Effect of RTIP on overall quality of life

**Figure 5:**
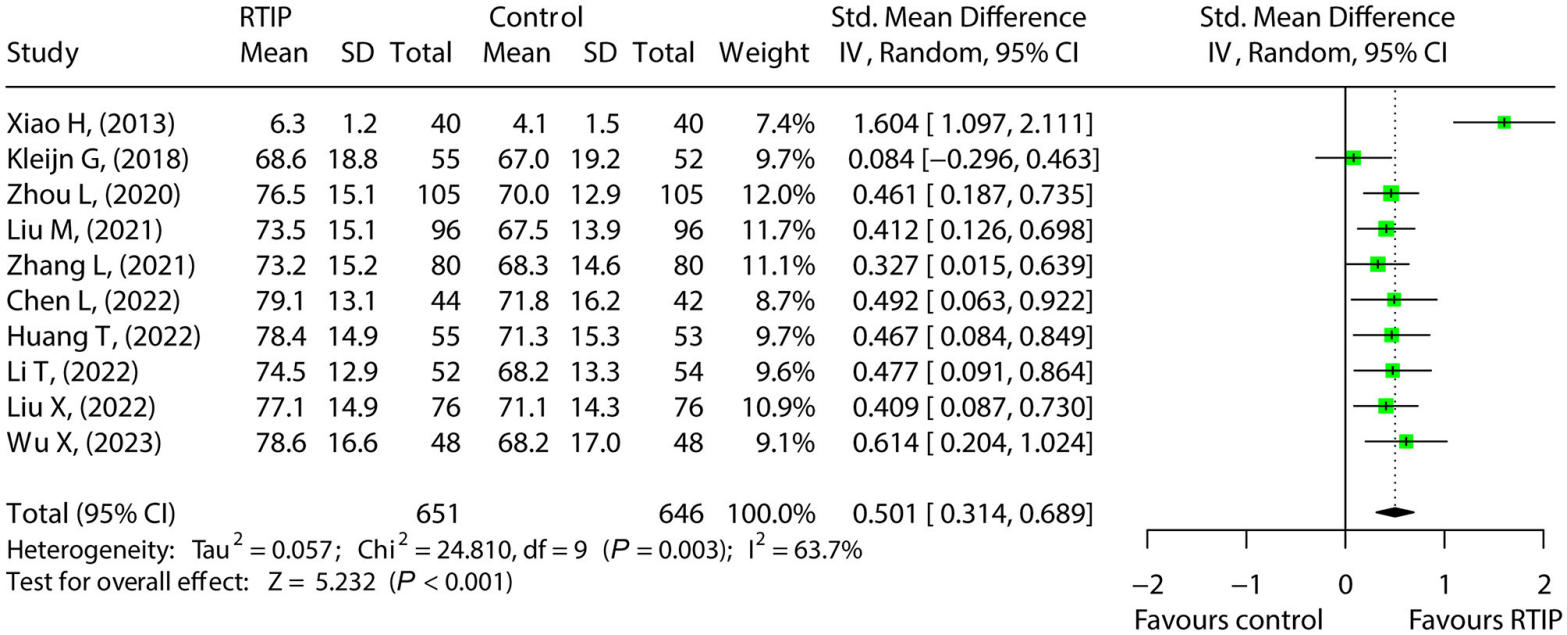
RTIP, with or without control care, elevates the overall quality of life of cancer patients.

A total of 10 studies compared the overall quality of life between the RTIP and control groups, where heterogeneity existed (*I*^2^ = 63.7%, *P* = 0.003). The random effects model disclosed elevated overall quality of life in the RTIP group compared to the control group [SMD (95% CI): 0.501 (0.314, 0.689), *P* < 0.001; Figure 5].

### 3.7 Further analysis

Respectively, 7, 4, 7, 4, and 4 studies compared anxiety score, anxiety rate, depression score, depression rate, and overall quality of life of digestive system cancer patients between the RTIP group and the control group. It was observed that in digestive system cancer patients, anxiety score [SMD (95% CI): −0.720 (−0.996, −0.444), *P* < 0.001], anxiety rate [RR (95% CI): 0.672 (0.522, 0.866), *P* = 0.002], depression score [SMD (95% CI): −0.997 (−1.625, −0.370), *P* = 0.002], and depression rate [RR (95% CI): 0.704 (0.548, 0.906), *P* = 0.006] was declined, and overall quality of life [SMD (95% CI): 0.451 (0.285, 0.617), *P* < 0.001] was increased in the RTIP group compared to the control group (Table 4).

**Table 4:** Meta-analysis for particular patients with digestive system cancers.

**Items**	**Number of studies**	**Selected model**	**Test for overall effect**		**Total effect (95% CI) (RTIP vs. control)**
			***Z*** **-value**	***P*** **-value**	
Anxiety score	7	Random	−5.112	<0.001	−0.720 (−0.996, −0.444)
Anxiety rate	4	Fixed	−3.068	0.002	0.672 (0.522, 0.866)
Depression score	7	Random	−3.114	0.002	−0.997 (−1.625, −0.370)
Depression rate	4	Fixed	−2.732	0.006	0.704 (0.548, 0.906)
Overall quality of life	4	Fixed	5.324	<0.001	0.451 (0.285, 0.617)

CI, confidence interval; RTIP, reminiscence therapy-involved program.

The particular patients with digestive system cancers combined types of colorectal cancer, gastric cancer, hepatocellular carcinoma, and digestive system cancer.

We have also conducted moderator analysis based on sessions of intervention and practice approach (Supplementary Table 2). The data showed that the RTIP group had lower anxiety scores, depression scores, and depression rates, as well as higher overall quality of life compared with the control group in studies with sessions of intervention ≤12 or >12. However, the anxiety rate was only lower in the RTIP group compared with the control group in studies with sessions of intervention >12. Regarding practice approach, both studies with hospital-based practice approach or others illustrated a lower anxiety score and higher overall quality of life in the RTIP group compared with the control group, while the depression score was only reduced in the RTIP group compared with the control group in studies with the hospital-based practice approach.

## 4 Discussion

Reminiscence therapy is a process in which individuals reflect on their lives and past experiences with the aid of music, photographs, videos, and other props. This therapy traces the core theory of “life review,” which can help promote a sense of integrity and adjustment (Zhong et al., 2023). As a non-pharmacological intervention, reminiscence therapy typically targets older adults with mental health difficulties.

However, its application in cancer patients has attracted increasing attention (Guo et al., 2022; Zheng et al., 2022; Wu and Zhang, 2023; Yan et al., 2023). For example, one study demonstrated that reminiscence therapy decreases both anxiety and depression scores in the first month and sixth months after intervention compared to routine care in patients with digestive system cancer (Zheng et al., 2022). Another study discloses reduced anxiety and depression in elderly lung cancer patients treated with reminiscence therapy compared with those receiving usual care (Guo et al., 2022). This meta-analysis found that RTIP with or without control care reduced the score and rate of anxiety and depression compared to control care in cancer patients. The probable explanations were as follows: (i) The procedure of RTIP involved the recall and sharing of selected personal memories and stories, which facilitated a more positive view of life (Kleijn et al., 2018). (ii) In addition, RTIP also drew past, present, and future together, which contributed to ego integrity and assisted patients in adapting to disease-related losses. (iii) During the process of RTIP, cancer patients perceived caring and support from the medical workers, which reduced their feelings of isolation (Laidlaw et al., 2023). Combining these aspects, RTIP with or without control care attenuated the score and rate of anxiety and depression compared to control care in cancer patients.

Impaired quality of life is another common concern in cancer patients (Suarez-Almazor et al., 2021). One previous study identifies that reminiscence therapy elevates the QLQ-C30 global health status score compared with usual control in recurrent gastric cancer patients (Wu and Zhang, 2023). Another clinical study noted that the overall quality of life does not vary between reminiscence therapy and usual care in cancer patients (Kleijn et al., 2018). The meta-analysis elucidated that RTIP with or without control care elevated the overall quality of life compared to control care in cancer patients. The possible reasons might be that symptoms of anxiety and depression adversely affect the overall quality of life (Polanski et al., 2018; van Montfort et al., 2020; Carbajal-Lopez et al., 2022). However, as described above, RTIP effectively alleviated anxiety and depression in cancer patients, which led to improved psychological wellbeing. Subsequently, the overall quality of life was improved in cancer patients who underwent RTIP.

Besides, it is worth discussing that RTIP can be conducted individually, one-on-one with a therapeutic listener, or in a group form, and the choice of modality is controversial. Some clinicians think that the treatment effect may be compromised in a group setting, as patients may not be willing to share their privacy and personal feelings with others (Ando et al., 2007). Conversely, other medical workers argue that RTIP in a group setting is beneficial for both patients and implementers. Patients can encourage each other and build closer social relationships, while the group setting allows for more efficient use of resources for the implementers compared to one-on-one sessions (Rubin et al., 2019).

Therefore, further research is needed to address these differing perspectives. Another important consideration is that implementing RTIP requires additional medical resources. Therefore, relevant studies are warranted to explore whether the clinical benefits of patients outweigh the medical costs of such interventions.

The current meta-analysis also conducted moderator analysis based on the sessions of intervention sessions (≤ 12 or >12) and the practice approach (hospital-based or others). The data suggested that the effect of RTIP on the anxiety score and overall quality of life was not affected by the number of intervention sessions or the practice approach. However, RTIP showed no effect on anxiety rate in studies with intervention sessions ≤12 and on depression scores in studies using non-hospital-based approaches. These findings indicate that RTIP might be more effective with intervention sessions >12 and a hospital-based approach. Therefore, clinicians might consider encouraging patients to undergo hospital-based RTIP with longer intervention sessions to maximize the effectiveness of this treatment.

A previous meta-analysis of RCTs revealed that reminiscence therapy reduced anxiety and depression scores while promoting overall quality of life in patients with cancers; however, this previous meta-analysis only included studies published before 2021 (Sun et al., 2023). Therefore, we conducted an updated meta-analysis and found similar findings: RTIP decreased anxiety and depression and improved the overall quality of life in cancer patients. Based on these meta-analyses, the application of RTIP could be recommended in cancer patients to improve psychological health and overall quality of life, especially using a hospital-based approach with longer intervention sessions. Despite the overall good quality of evidence and the absence of no publication bias in each outcome, some limitations should be mentioned. First, blinding was difficult to implement, resulting in a high risk or unclear risk of performance bias in the included studies. Second, the sample size of most included studies was relatively small.

Consequently, larger-scale studies should be conducted to verify these findings. Third, due to the limited amount of evidence, the influence of some particular intervention details, such as RTIP session compositions, length, and frequency, on treatment efficacy could not be evaluated in this meta-analysis. Fourth, due to the limited number of studies, subgroup analysis in patients with cancers other than the digestive system could not be performed. Future studies should investigate the effects of RTIP in patients with other types of cancers. Fifth, articles published in other languages might have been excluded. Further studies could consider using artificial intelligence to bridge the language gap. Finally, since the data based on age, sample size, and different reminiscence content could not be combined, a moderator analysis based on these variables could not be performed.

In summary, RTIP efficiently improves the psychological health and overall quality of life in cancer patients. This therapy can be included in oncology care plans to meet multidimensional care needs and enhance the general health condition of cancer patients. Future studies should consider investigating whether different materials used in the intervention, different facilitators, and the frequency of intervention could affect the outcomes of RTIP in cancer patients.

## Data availability statement

The original contributions presented in the study are included in the article/Supplementary material, further inquiries can be directed to the corresponding author.

## Author contributions

XS: Data curation, Investigation, Methodology, Writing – original draft. WS: Data curation, Formal analysis, Methodology, Visualization, Writing – original draft. MY: Data curation, Formal analysis, Methodology, Resources, Writing – original draft. LX: Conceptualization, Resources, Supervision, Validation, Writing – review & editing.
