# Supplementary material for: Effects of a reminiscence therapy-involved program on anxiety, depression, and the quality of life in cancer patients: a meta-analysis of randomized controlled trials
Source: Front Psychol. 2024 Sep 17;15:1408941. doi: 10.3389/fpsyg.2024.1408941 (PMC11443599; doi:10.3389/fpsyg.2024.1408941)
Supplement: Supplementary file 1 [file Data_Sheet_1.docx]

***Supplementary Materials***

**Supplementary Table 1.** Quality assessment via Cochrane risk of bias 2.0.

| Studies | Domain 1 | Domain 2 | Domain 3 | Domain 4 | Domain 5 | Overall |
| --- | --- | --- | --- | --- | --- | --- |
| Xiao H, (2013) (19) | Low risk | Low risk | Low risk | Low risk | Low risk | Low risk |
| Vuksanovic D, (2016) (20) | Low risk | Low risk | Low risk | Low risk | Low risk | Low risk |
| Kleijn G, (2018) (21) | Low risk | Low risk | Low risk | Low risk | Low risk | Low risk |
| Dong X, (2019) (22) | Low risk | Low risk | Low risk | Low risk | Low risk | Low risk |
| Zhou L, (2020) (23) | Low risk | Low risk | Low risk | Some concerns | Low risk | Some concerns |
| Liu M, (2021) (24) | Low risk | Low risk | Low risk | Some concerns | Low risk | Some concerns |
| Zhang L, (2021) (25) | Low risk | Low risk | Low risk | Some concerns | Low risk | Some concerns |
| Zhao X, (2021) (26) | Low risk | Low risk | Low risk | Some concerns | Low risk | Some concerns |
| Chen L, (2022) (27) | Low risk | Low risk | Low risk | Some concerns | Low risk | Some concerns |
| Guo Q, (2022) (28) | Low risk | Low risk | Low risk | Some concerns | Low risk | Some concerns |
| Huang T, (2022) (29) | Low risk | Low risk | Low risk | Some concerns | Low risk | Some concerns |
| Li T, (2022) (30) | Low risk | Low risk | Low risk | Some concerns | Low risk | Some concerns |
| Liu X, (2022) (31) | Low risk | Some concerns | Low risk | Some concerns | Low risk | Some concerns |
| Zheng M, (2022) (32) | Low risk | Some concerns | Low risk | Low risk | Low risk | Some concerns |
| Babaei N, (2023) (33) | Low risk | Some concerns | Low risk | Low risk | Low risk | Some concerns |
| Wu X, (2023) (34) | Low risk | Low risk | Low risk | Some concerns | Low risk | Some concerns |

Domain 1: bias arising from the randomization process;

Domain 2: bias due to deviations from intended interventions;

Domain 3: bias due to missing outcome data;

Domain 4: bias in measurement of the outcome;

Domain 5: bias in selection of the reported result.

**Supplementary Table 2.** Moderator analysis.

| Subgroups | Number of studies | Selected model | Test for overall effect | | Weight | Subtotal effect (95% CI) |
| --- | --- | --- | --- | --- | --- | --- |
|  |  |  | Z value | *P* value |  |  |
| **Anxiety score** |  |  |  |  |  |  |
| Sessions of intervention |  |  |  |  |  |  |
| ≤12 | 7 | Random | -3.991 | <0.001 | 45.3% | -0.680 (-1.014, -0.346) |
| >12 | 7 | Random | -8.232 | <0.001 | 54.7% | -0.502 (-0.622, -0.383) |
| Practice approach |  |  |  |  |  |  |
| Hospital-based | 12 | Fixed | -10.094 | <0.001 | 85.1% | -0.517 (-0.617, -0.416) |
| Others | 3 | Fixed | -5.332 | <0.001 | 14.9% | -0.652 (-0.892, -0.412) |
| **Anxiety rate** |  |  |  |  |  |  |
| Sessions of intervention |  |  |  |  |  |  |
| ≤12 | 3 | Fixed | -1.790 | 0.073 | 19.6% | 0.712 (0.491, 1.033) |
| >12 | 7 | Fixed | -3.265 | 0.001 | 80.4% | 0.742 (0.621, 0.888) |
| **Depression score** |  |  |  |  |  |  |
| Sessions of intervention |  |  |  |  |  |  |
| ≤12 | 7 | Random | -3.006 | 0.003 | 48.6% | -1.022 (-1.689, -0.356) |
| >12 | 7 | Random | -7.093 | <0.001 | 51.4% | -0.431 (-0.550, -0.312) |
| Practice approach |  |  |  |  |  |  |
| Hospital-based | 12 | Random | -3.675 | <0.001 | 80.4% | -0.611 (-0.937, -0.285) |
| Others | 3 | Random | -1.808 | 0.071 | 19.6% | -0.896 (-1.868, 0.075) |
| **Depression rate** |  |  |  |  |  |  |
| Sessions of intervention |  |  |  |  |  |  |
| ≤12 | 3 | Fixed | -2.597 | 0.009 | 19.5% | 0.588 (0.394, 0.878) |
| >12 | 7 | Fixed | -4.589 | <0.001 | 80.5% | 0.642 (0.532, 0.776) |
| **Overall quality of life** |  |  |  |  |  |  |
| Sessions of intervention |  |  |  |  |  |  |
| ≤12 | 5 | Random | 2.792 | 0.005 | 44.6% | 0.635 (0.189, 1.081) |
| >12 | 5 | Random | 5.875 | <0.001 | 55.4% | 0.414 (0.276, 0.553) |
| Practice approach |  |  |  |  |  |  |
| Hospital-based | 8 | Fixed | 7.290 | <0.001 | 86.7% | 0.443 (0.324, 0.562) |
| Others | 2 | Fixed | 4.062 | <0.001 | 13.3% | 0.629 (0.326, 0.933) |

CI, confidence interval.
